# TSPO Ligand 2-Cl-MGV-1 Mitigates Traumatic Brain Injury (TBI) in a Mouse Model

**DOI:** 10.3390/ijms26104854

**Published:** 2025-05-19

**Authors:** Nasra Yasin, Leo Veenman, Beatriz Caballero, Nidal Zeineh, Laura Gonzalez-Blanco, Abraham Weizman, Moshe Gavish

**Affiliations:** 1 The Ruth and Bruce Rappaport Faculty of Medicine, Technion—Israel Institute of Technology, Haifa 31096, Israel; nasra.yassin.1991@gmail.com (N.Y.); jehudaveenman@gmail.com (L.V.);; 2 Department of Morphology and Cell Biology, Faculty of Medicine, University of Oviedo, 33006 Oviedo, Spain; caballerobeatriz@uniovi.es (B.C.); laurablanco94@hotmail.com (L.G.-B.); 3 Instituto de Neurociencias del Principado de Asturias (INEUROPA), 33003 Oviedo, Spain; 4 Instituto de Investigación Sanitaria del Principado de Asturias (ISPA), 33011 Oviedo, Spain; 5 Servicio Regional de Investigación y Desarrollo Agroalimentario (SERIDA), 33300 Villaviciosa, Spain; 6 Faculty of Medical and Health Sciences, Tel Aviv University, Tel Aviv 6997801, Israel; weizmana@gmail.com; 7 Laboratory of Molecular and Biological Psychiatry, Felsenstein Medical Research Center, Petah Tikva 4910002, Israel; 8 Research Unit, Geha Mental Health Center, Petah Tikva 4910002, Israel

**Keywords:** translocator protein (TSPO), mitochondria, gene expression, neurodegeneration, brain damage, magnetic resonance imaging (MRI)

## Abstract

In this study, we assessed the ability of 2-Cl-MGV-1 (2-chlorophenyl quinazolin-4-yl, dimethyl carbamate), a ligand of the 18 kDa mitochondrial translocator protein (TSPO), to mitigate brain damage in a mouse model of traumatic brain injury (TBI). TSPO is important for arresting the death of neurons and glia and counteracting microglial activation, and it provides anti-inflammatory activity, promotes regeneration (including neurons), and contributes to angiogenesis. We assessed the minimal dose of the TSPO ligand 2-Cl-MGV-1 that attenuates the magnitude of brain damage as well as the time window following TBI in which the treatment is effective. We found that 7.5 mg/kg of 2-Cl-MGV-1 can reduce the impact of the TBI as assessed by magnetic resonance imaging (MRI). We also found that 2-Cl-MGV-1 improved motor performance as observed in a treadmill test (80.9% fewer shocks needed and 40.7% more distance covered, both *p* < 0.05), and reduced anatomical brain damage (by 86.5%, *p* < 0.05), cell death (by 75.0%, *p* < 0.001), and microglial inflammatory response (by 50.2%, *p* < 0.01). The treatment also increased expression of neuronal markers NeuN and β3-tubulin (30.0%, *p* < 0.01; 36.0%, *p* < 0.01, respectively). The time window in which we found the treatment to be effective was 3–11 h after TBI. Our study suggests that agents active at the TSPO can significantly attenuate the outcome of TBI, including in the structural, cellular, and neuro-behavioral dimensions. The mechanisms involved in the attenuation of brain damage following TBI may be related to a decrease in cell death and to anti-inflammatory activity. TSPO seems to be a novel target for the development of agents aimed at the suppression of neurodegenerative processes.

## 1. Introduction

Traumatic brain injury (TBI) presents a complex clinical picture with a broad spectrum of symptoms and disabilities [1,2]. It is the leading cause of death among people aged 45 years or younger, with a mortality rate of 40% [3]. TBI resulting from falls causes a high rate of death and hospitalization among people aged 75 years or older [4]. TBI involves chronic microglial activation and neuronal and astrocytic cell death [5].

TSPO ligands halt microglia activation and promote neurodifferentiation, axon regeneration, stem cell activation, and angiogenesis and may thus also attenuate TBI impact [6]. TBI is associated with brain edema as well as with skull fracture, bleeding, and inflammation. Current treatment is restricted to neurosurgical intervention [5]. Evidence shows that as soon as within the first day of injury, human TBI is associated with changes in the expression of approximately 7000 genes in the brain area surrounding the primary injury [7]. Similar changes to the expression of genes have also been shown in animal models following TBI. TSPO ligands have been shown to counteract the effects of TBI. TSPO knockdown modulates functional pathways that are affected in animal TBI [5]. Thus, it seems that TSPO is relevant to the pathophysiology and pharmacotherapy of TBI.

In experimental models, ligands of TSPO have been found to reduce pro-inflammatory gene expression of COX-2, TNF-α, and IL-6 after being stimulated with lipopolysaccharide (LPS) [6]. TSPO has thus become the target of research into high-affinity therapeutic ligands aimed at treating various pathologies of the brain [8]. It has been suggested that relatively high concentrations (typically higher than 50 µM) of high-affinity TSPO ligands often induce programmed cell death, while relatively low concentrations, even in the nM range, protect against induction of programmed cell death [9,10]. In contrast, it has been found that low- to moderate-affinity TSPO ligands, such as etifoxine, can ameliorate the effects of brain injury and brain disorders [11,12].

The contribution of TSPO to the arrest of neuron and glia cell death is mediated by intracellular mechanisms that affect ATP production, reactive oxygen species (ROS) generation, Ca^2+^ release, and cytochrome c release from the mitochondria, leading to programmed cell death [13]. TSPO also regulates inflammatory responses [12].

In the present study, we used the low-affinity TSPO ligand 2-Cl-MGV-1 (in contrast to the classical high-affinity TSPO ligands such as PK 11195, Ro5 4864, and FGIN-1-27) at relatively high doses to treat a mouse model of TBI. We chose this low-affinity TSPO ligand to avoid the possible negative side effects of high-affinity TSPO ligands, including cell death caused by relatively high doses [9,14]. The study assessed the impact of the ligand on TBI in mice by assessing changes in brain lesions over time and improvement in motor deficits following the TBI. The status of the brain damage was evaluated by assessing the neuroanatomical and neurohistological changes, including immunohistochemistry.

## 2. Results

Experiments 1 and 2 show that 2-Cl-MGV-1 administration (5 days/week and 7 days/week, respectively) ameliorates the size of the brain damage induced by TBI. The advantage of Experiment 2 was in the division of the mice into two groups with similar distribution of brain damage size. Experiment 3 further supported the results of Experiment 2 by showing significant improvements in the behavioral treadmill tests in the mice treated with 2-Cl-MGV-1 as compared to those treated with DSMO and untreated mice.

### 2.1. Experiment 1

**Figure 1:**
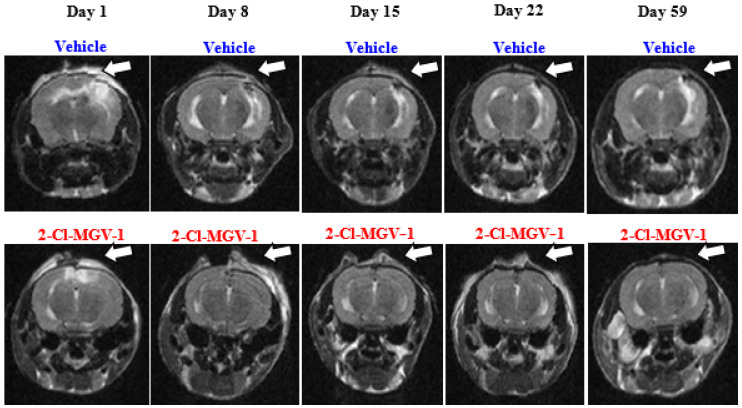
(Experiment 1): Representative MRI scans of TBI-induced brain damage in mice, throughout the study period, treated with vehicle or 2-Cl-MGV-1 (*n* = 6 per group). The white arrow points at the damaged area. **Top row**: TBI-induced damage shrinks less when treated with vehicle (DMSO). On day 1, the injured brain area is white, indicating hemorrhaging or edema. On day 8, the white area is reduced, and the injured tissue appears fragmented, within a black area. On days 15, 22, and 59, the injured brain tissue disappears, but there is still a white area in the proximity of the damaged tissue, and it is larger on day 59. The lesioned tissue detected by MRI on day 59 can also been seen with microscopy, as shown in Figure 2. **Bottom row**: TBI-induced damage almost disappears when treated with 7.5 mg/kg of 2-Cl-MGV-1. On day 8, an already-significant reduction in injury size can be seen, which continues into the following weeks, and by day 59, it is nearly undiscernible by MRI.

As can be seen in representative Figure 1, the vehicle by itself (DMSO administered 5 days/week for 60 days) was found to have no effect on the brain injury volume (whose size immediately after TBI averaged 7.3 ± 2.2 mm^3^). Additionally, 2-Cl-MGV-1 (7.5 mg/kg) administration that started 3–7 h after TBI and was administered 5 days/week, reduced the size of brain injury (whose size immediately after TBI averaged 10.7 ± 3.1 mm^3^) significantly already 1 week after TBI, and the beneficial effect persisted throughout the 59-day study period. This pilot study suggests that 2-Cl-MGV-1 can reduce brain damage caused by TBI.

**Figure 2:**
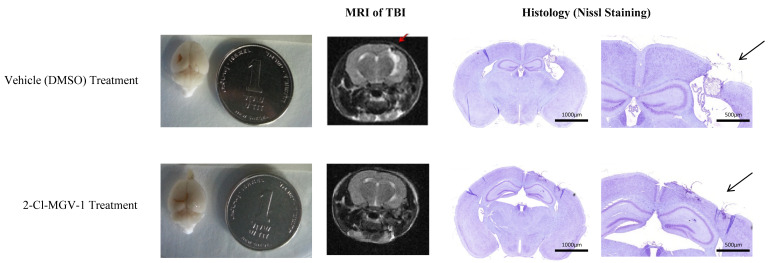
(Experiment 1). Histological and MRI evaluations of TBI extent (representative). Arrows point to damaged areas. After 59 days of 2-Cl-MGV-1 (7.5 mg/kg) and vehicle (DMSO) treatments, given 5 days per week, TBI volume was evaluated with MRI and by histological Nissl staining. Injury size in the ligand (2-Cl-MGV-1)-treated brains (**bottom row**) was significantly smaller than in the vehicle (DMSO)-treated brains (**top row**). **Top row**: The black spot pointed to by the red arrow is a hole in the brain seen in the Nissl-stained section. The relatively small conspicuous white spot under the red arrow is scar tissue. The large, light purple area shows enlarged lateral ventricles. In the **bottom row**, the MRI does not show aberrations from normal brain tissue. However, in the histological sections, we see shrinkage of cortical tissue at the site of the original injury. This we do not see in the corresponding MRI scan of the live mouse. This suggests that the shrinkage occurred postmortem, probably during the dehydration series. MRI does not show damage at all levels. Thus, in our case, the injury caused there to be fewer cells in the injured area, replacing cells with CSF, and the dehydration process during the histological procedure made the damage visible.

Figure 2 shows representative MRI images, where white areas (hot spots) relate to edema or bleeding, and black areas (lesions) relate to tissue damage (holes). The MRI results were corroborated by histological microscopy studies. Representative images of the histological studies (Figure 2) show MRI images of the “hot spots” and “lesions” in histological sections observed under the microscope. Typically, a lesion (black area) is a hole in the brain. Hot spots (white areas associated with the TBI impact site in an otherwise grey brain area) indicate excess fluid, either edema, bleeding, or CSF, usually with enlarged ventricles [15].

As can be seen in Figure 1 and Figure 2, significant effects of 2-Cl-MGV-1 on the injured area were found with both MRI and histology.

### 2.2. Experiment 2

In this experiment, we used the learnings from Experiment 1 to improve the procedures, thus overcoming various technical problems and reducing undesired variability in the results. The improvements are described later in the weight drop apparatus and TBI subsection of Section 4.

#### Neurohistological Results, Experiment 2, Young Mice (~20 gr)

**Figure 3:**
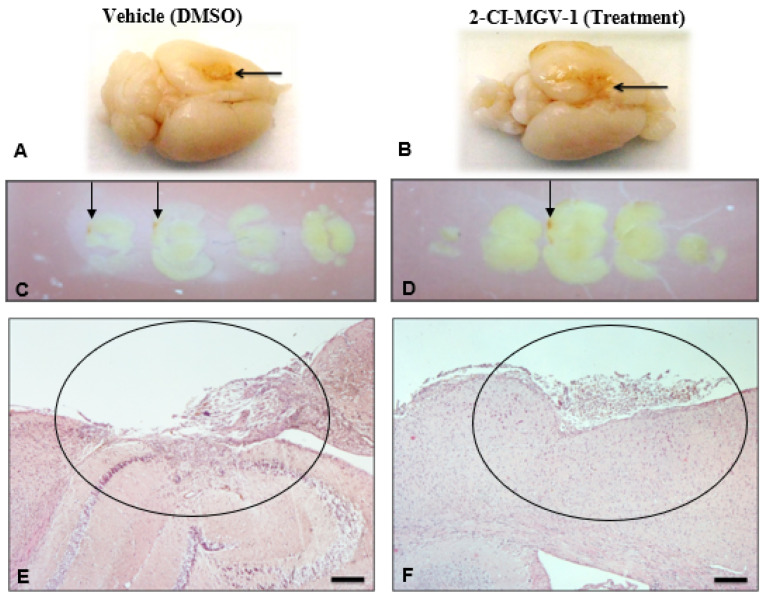
(Experiment 2). Representative histological images demonstrating the therapeutic effect of 2-Cl-MGV-1 on the TBI. In all the mouse brains, the damage was in the parietal and frontal lobes. (**A**,**C**,**E**) show the brain of a vehicle (DMSO)-treated mouse. (**B**,**D**,**F**) show the brain of a 2-Cl-MGV-1 (7.5 mg/kg)-treated mouse. Both treatments were administered daily for 60 days (starting on TBI day). In (**A**,**B**), arrows point to the TBIs, which appear as brown areas. (**C**,**D**) show four and five blocks, respectively, cut from the paraffin-embedded brain sections of representative TBI mice. The arrows point to the injuries resulting from the TBI. (**E**,**F**) Histological sections of the representative brains stained with hematoxylin/eosin after the 60-day treatment period. The black circles indicate the injured areas. Initially, the injury size of the two displayed brains was similar—approximately 36 mm^3^. (**E**) A massive loss of brain tissue was detected in the left parietal cortex (circled by the ellipse) in the microscopic section of the vehicle (DMSO)-treated mouse with TBI. The injury crossed the corpus callosum, down into the hippocampus, and the granule neuron layer of the hippocampus was lost in the injured area. (**F**) The microscopic section of the 2-Cl-MGV-1-treated mouse with TBI shows a lower extent of damage, with less significant lesions than in the vehicle-treated mouse brain. In this brain, no conspicuous tissue disorganization can be seen beyond the minor lesions. Microscope photos were taken at 40× magnification. Scale bars = 200 μm.

Figure 3 shows representative examples of TBI mouse brains treated with vehicle (DMSO) or 7.5 mg/kg of 2-Cl-MGV-1 for 60 days (Figure 3A and Figure 3B, respectively). The efficacy of 2-CI-MGV-1 treatment for TBI in the mice can be seen at both macroscopic and microscopic levels. The injured areas, i.e., the brown areas in both examples of TBI presented in Figure 3A,B, typically are localized in the frontal and parietal lobes. Representative brains were prepared for macroscopic and microscopic observation as described in Section 4. In the macroscopic images (Figure 3C,D), the injured areas are pointed to by arrows. In the histological brain sections (Figure 3E,F), a massive loss of brain tissue can be observed in the vehicle-treated TBI brain (Figure 3E) at the left parietal cortex, even crossing the corpus callosum into the hippocampus, since the granule neurons layer of the hippocampus has also disappeared. The histological section of the 2-Cl-MGV-1-treated TBI brain shows good recovery from the damage (Figure 3F). We can see that loss of neural tissue in the cortex, after 2-Cl-MGV-1 treatment, is smaller compared to the vehicle-treated TBI brain with much of the neocortical layer recovered. The encircled areas mark the remaining brain damage following both treatments (Figure 3E,F).

**Figure 4:**
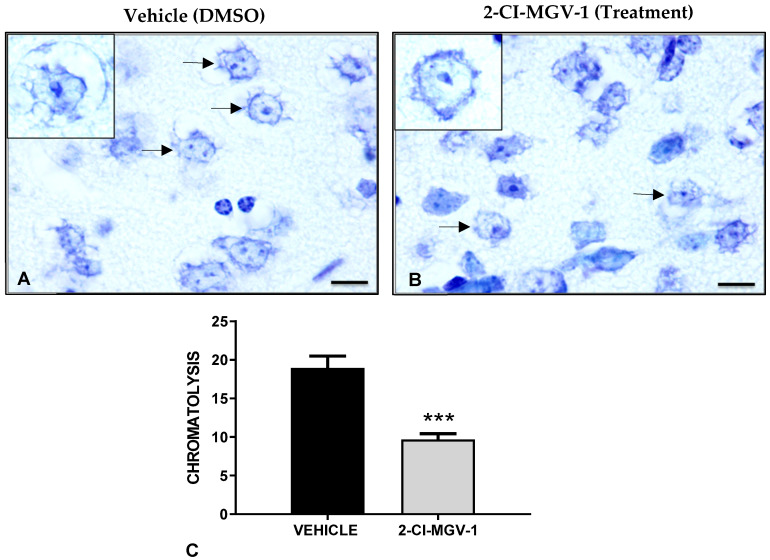
(Experiment 2): Representative histological images of mouse brains that underwent TBI showing Nissl staining and chromatolysis. Higher levels of chromatolysis indicate more severe injury resulting from TBI. Results from 2-Cl-MGV-1 administered for 60 days showed smaller damage compared to 60-day vehicle treatment of brain tissue. The arrows point to cells under chromatolysis. (**A**,**B**) Microscopic sections with Nissl body stain of vehicle-treated TBI brain (**A**) and 2-Cl-MGV-1-treated TBI brain (**B**) are shown. Loss and dispersion of the Nissl staining in the neuronal cytoplasm represents chromatolysis. In image (**A**)—the vehicle—the blue spots are clearly lighter than in image (**B**)—2-Cl-MGV-1—indicating more Nissl loss and dispersion, i.e., (**B**) shows less damage (chromatolysis) compared to the vehicle (DMSO)-treated brain. (**C**) Quantification of the chromatolysis in the mouse brains that underwent TBI following 60 days of treatment with either vehicle (**black**) or 2-Cl-MGV-1 (**grey**). Chromatolysis was assessed by counting cells negative for Nissl stain in three different brain fields in the neocortex close to the injured area of each mouse. The 2-Cl-MGV-1-treated brains show significantly less chromatolysis as compared to vehicle-treated brains. Data are represented as mean ± SEM. *** *p* < 0.001 after unpaired *t*-test. Micrographs were taken at 400× magnification, except small boxes, which were taken at 1000× magnification. N = 6 in each group. Scale bars = 20 μm.

The neurodegeneration associated with TBI can be seen in the disappearance of Nissl body labeling of neuronal cytoplasm (Figure 4). Nissl bodies were stained in sections of TBI brains of vehicle-treated (Figure 4A) and 2-Cl-MGV-1-treated mice (Figure 4B). The presence of chromatolysis around the brain injury area is evidenced by the scarcity of Nissl body stain in the representative brain of the vehicle-treated TBI mice (Figure 4A). We can observe, however, an abundance of the Nissl body stain in the representative brain of the 2-Cl-MGV-1-treated TBI mice (Figure 4B) compared to the vehicle-treated brain. Quantification of cells negative for Nissl stain reveals that the 2-Cl-MGV-1-treated TBI brain shows significantly less (*p* < 0.001) chromatolysis as compared to vehicle-treated TBI brains (Figure 4C).

**Figure 5:**
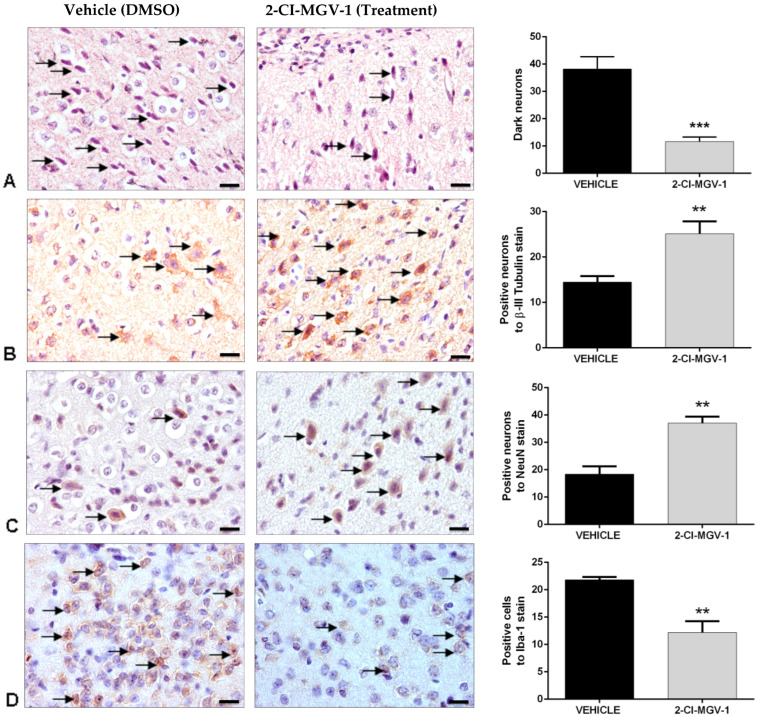
(Experiment 2). Representative immunohistological samples demonstrating a decrease in neurodegeneration and inflammation in the brains of TBI mice treated with 2-Cl-MGV-1 for 60 days compared to the brains of vehicle-treated TBI mice. Mice were treated with 7.5 mg/kg of 2-Cl-MGV-1 (in vehicle) or with vehicle alone for 60 days. In each microscopic image, arrows show cells positive for specific staining. (**A**) Dark neurons (degenerating neurons), stained with hematoxylin and eosin, in a representative vehicle-treated TBI sections and in a representative 2-Cl-MGV-1 treated TBI sections. The bar chart shows the analysis of the number of damaged neurons in three different fields in the cortices, close to the injury sites of the mice. The ligand-treated TBI mouse-brain shows significantly fewer (*p* < 0.001) damaged neurons than the vehicle-treated one. (**B**) Examples of immunohistochemical staining of β-III-Tubulin in the brain of a vehicle-treated TBI mouse and in the brain of a ligand-treated TBI mouse. The bar chart shows the number of neurons positive for β-III-Tubulin stain in three different fields in the cortex close to the injury sites of the mice. The ligand-treated TBI mouse brain shows significantly more (*p* < 0.01) β-III-Tubulin labeling as compared to the vehicle-treated TBI mouse brain. (**C**) Examples of NeuN immunohistochemical labeling in a vehicle-treated TBI mouse brain and in a ligand-treated TBI mouse brain. The bar chart shows the number of NeuN-positive neurons in three different fields of the cortex close to the injury sites of the mice. The ligand-treated TBI mouse brain shows significantly more (*p* < 0.01) NeuN staining as compared to the vehicle-treated TBI mouse brain. (**D**) Examples of Iba-1 immuno-positive cells in a vehicle-treated TBI mouse brain and a ligand treated TBI mouse brain. The bar chart shows analysis of the number of Iba-1 positive cells in 3 different fields of the cortex close to the injury sites of the mice. The 2-Cl-MGV-1-treated TBI mouse-brain shows a significantly lower (*p* < 0.01) number of Iba-1 positive cells as compared to the vehicle-treated TBI mouse-brain. Micrographs were taken at 400× magnification. The scale bars measure 20 µm. N = 6 in each group. Data are represented as mean ± SEM. ** *p* < 0.01; *** *p* < 0.001.

Neurodegeneration in TBI brains was analyzed by the specific staining of dark neurons (degenerating neurons) with hematoxylin and eosin (Figure 5A) as well as with immunohistochemical staining of the neuronal markers β-III-Tubulin and NeuN (Figure 5B and Figure 5C, respectively). It can be seen that the brains of 2-Cl-MGV-1-treated TBI mice show significantly fewer (*** *p* < 0.001) dark neurons as compared to the brains of vehicle-treated TBI mice (Figure 5A). Similarly, the brains of TBI mice treated with 2-Cl-MGV-1 for 60 days show significantly higher (** *p* < 0.01) levels of the neuronal markers β-III-Tubulin and NeuN (Figure 5B and Figure 5C, respectively) as compared to the brains of vehicle TBI mice (Vehicle). Finally, microglial activation was assessed by immunohistochemical staining with Iba-1, a marker for reactive microglia (Figure 5D). TBI brains of 2-Cl-MGV-1-treated mice show significantly lower levels (** *p* < 0.01) of Iba-1 labeling as compared to the brains of vehicle-treated TBI mice (Figure 5D).

In conclusion, these histological results (Figure 2, Figure 3, Figure 4 and Figure 5) show a reduction in neuronal death, increase in neuronal number, increase in marker for neurons, and reduction in number of activated glia. Treatment with 2-Cl-MGV-1 for 60 days can significantly reduce neurodegenerative damage and microglia activation characteristic in brains of mice with TBI.

### 2.3. Experiment 3

In this experiment, a new cohort of twenty-nine *C57BL/6N* mice was divided into the following groups: (1) naïve, i.e., no TBI and no treatment (N = 5); (2) TBI with no subsequent treatment (untreated; N = 10); (3) TBI with subsequent vehicle-only (DMSO) treatment (TBI + vehicle; N = 7); (4) TBI with subsequent 2-Cl-MGV-1 treatment (TBI + Ligand; N = 7). The first treatment in the TBI + vehicle and the TBI + ligand groups was administered 4.5–5.5 h after TBI and daily thereafter for another 59 days. The brain injury sizes were measured by MRI approximately 1 h post TBI, as well as on day 1, day 8, day 15, day 22, and day 59 after the TBI. The motor and learning capabilities of the mice were assessed with the treadmill apparatus at the following time points: day 1, day 8, day 15, and day 22 after the TBI.

**Figure 6:**
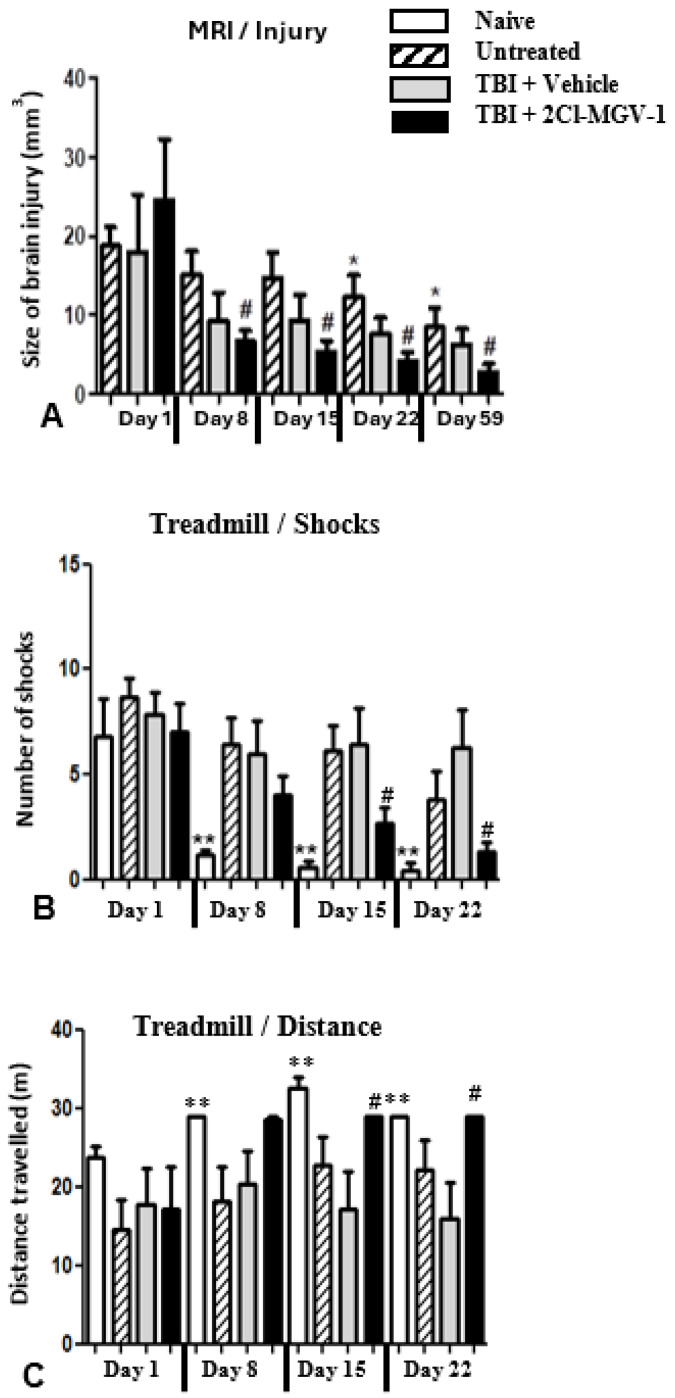
(Experiment 3). Effects of 2-Cl-MGV-1 treatment on brain injury volume and treadmill performance after TBI induction, compared to vehicle-treated and untreated mice and to naïve mice (no TBI and no treatment). Naive—no TBI and no treatment; TBI untreated—TBI and no treatment whatsoever; TBI + vehicle—TBI and vehicle (DMSO) treatment; TBI + ligand—TBI and 2-Cl-MGV-1 treatment. # *p* < 0.05 vs. TBI + ligand on day 1, * *p* < 0.05 vs. untreated on day 1; ** *p* < 0.01 vs. naïve on day 1. (**A**–**C**)—For all, weekly measurements are compared to the measurements taken immediately after TBI induction. (**A**) The TBI + ligand group showed significant reductions in brain injury volume every week, starting from day 8. The untreated group also showed significant improvement, but theirs started only on day 22 after TBI induction. (**B**) Naïve mice improved their performance very significantly (avoiding footshocks on a treadmill apparatus) as early as day 8 (after the other groups’ TBI induction), and improvement continued throughout day 22. The TBI + ligand group started improving their performance significantly on day 15 after TBI induction and continued improving through day 22. The TBI + vehicle group did not show improvement in avoiding shocks on the treadmill. Untreated mice showed some improvement on day 22, but this did not reach statistical significance. (**C**) While the naïve group performed best by far on this test (as expected), the TBI + ligand group also showed good improvement that gained statistical significance on day 15. Statistical analysis was conducted using RM-ANOVA followed by one-way ANOVA and by Mann–Whitney post hoc test.

Figure 6 shows the beneficial effects of 2-Cl-MGV-1 on the size of the TBI and on the treadmill performance. Figure 6A demonstrates the shrinkage in TBI size over the 60-day period after injury, as demonstrated by MRI. MRI analysis of the volume of the brain injury was monitored on the day of TBI induction and then on days 8, 15, 22, and 59 after the TBI. On the day of injury, the three groups that underwent TBI have similar average TBI sizes.

All three groups demonstrated some reduction in average injury size throughout the 60-day experiment duration; however, it appears that the TBI + ligand group showed the biggest reduction. Additionally, starting on day 8 and continuing through day 59 after TBI, the mice in the TBI + ligand group displayed a significant continuing reduction in injury volume compared to the average injury size of the group on day 1 (Figure 6).

The TBI + vehicle group did not demonstrate significant reduction in the average injury size throughout the 60-day experiment, while the untreated group showed a significant reduction only starting on day 22.

Figure 6B,C shows better performance on the treadmill during the experiment period by mice treated with 2-Cl-MGV-1 than by the DMSO-treated mice. Figure 6B presents the average number of shocks per session received by each group of mice. In other words, it shows the ability of the mice to continue running on the treadmill, thus avoiding the electric shocks administered to their feet.

As expected, the group that performed best on this test was the naïve group, but it was followed by the TBI + ligand group. In the latter, the improvement in performance compared to that of day 1 became significant on day 22 and continued to day 59. While there was some improvement also in the other two groups, it never reached significance.

Figure 6C shows that the TBI + ligand group performed significantly better than the other two TBI groups on the distance facet of the treadmill, improving the distance every week, and the improvement reached significance on day 22. Here again, unsurprisingly, the naïve group performed best.

In conclusion, as can be seen in Figure 6A–C, 2-Cl-MGV-1 shows positive effects when administered after TBI in all three parameters: brain injury size measured with MRI, behavioral performance tested by treadmill shocks, and treadmill distance measured in meters.

These findings are consistent with our histochemical results, as described earlier in the manuscript that points to 2-Cl-MGV-1 as an option for treatment of TBI.

Importantly, our assays of blood serum, described below in Experiment 4, did not indicate liver or kidney damage due to 2-Cl-MGV-1 in DMSO, neither did DMSO by itself.

### 2.4. Experiment 4

**Figure 7:**
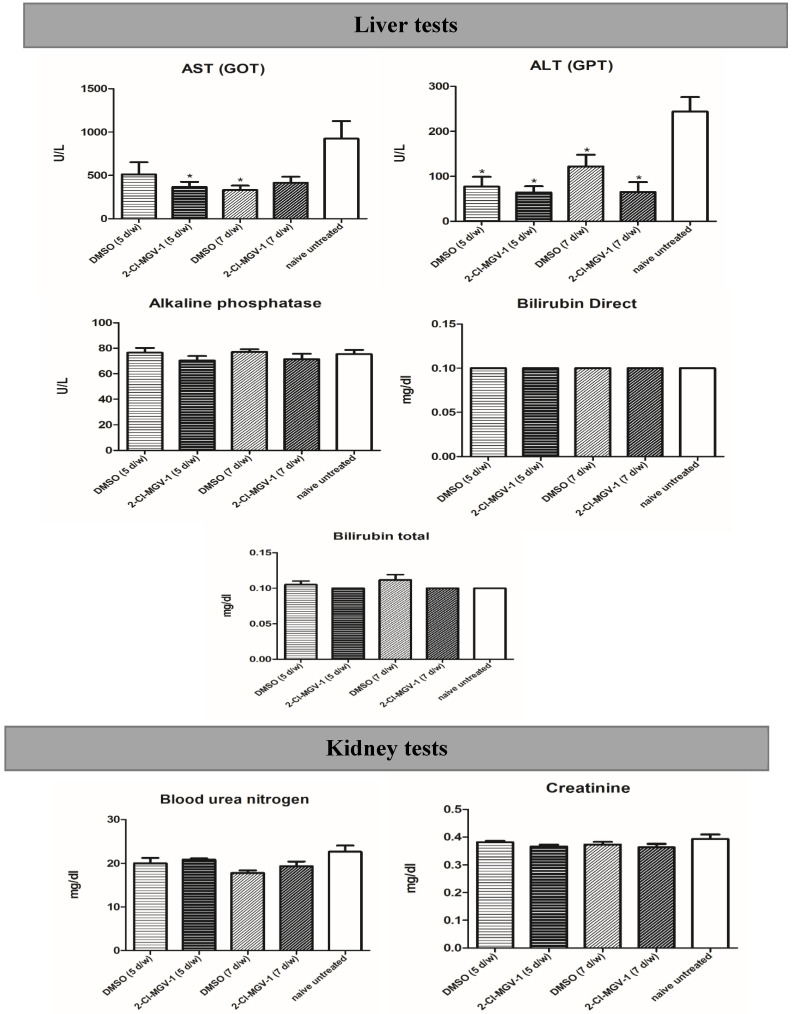
Effects of 60 days of 5- and 7-day-per-week treatment with DMSO or 2-Cl-MGV-1 in mice that had not undergone traumatic brain injury as compared to a parallel untreated control group (naïve, i.e., without any injection). Liver and kidney values measured: AST(GOT), ALT(GPT), alkaline phosphatase, bilirubin direct, bilirubin total, urea nitrogen, and creatinine. Briefly, the liver and kidney values of the experimental groups either did not show significant differences compared to either naïve untreated or reductions. Results presented as means ± SEM. Two-tailed ANOVA, (* = *p* < 0.05 versus naive) *n* = 6 in each group.

To check the safety of 2Cl-MGV-1, we assessed liver and kidney function using blood serum, as described in Section 4. As can be seen in Figure 7, we did not find adverse effects when comparing 2-Cl-MGV-1 treatment, vehicle treatment, and naïve control. The liver values of alkaline phosphatase, direct bilirubin and total bilirubin, and the kidney values of urea nitrogen and creatinine did not show any significant differences. The AST(GOT) and ALT(GPT) values for the experimental groups, while showing significant differences from the naïve, were all still in the normal range (namely, not higher than the naïve).

## 3. Discussion

The major finding of the current study is that the TSPO ligand 2-Cl-MGV-1 can reduce brain damage caused by TBI. Previous studies showed that 2-Cl-MGV-1 was efficacious in various models for neurodegeneration in in vitro cell culture models as well as in in vivo rodent models [5,10,14]. In addition, our studies in cell culture addressing gene expression relevant to various aspects of brain damage indicated that TSPO ligands, including 2-Cl-MGV-1, may have beneficial effects [16,17]. In the present study Mice were chosen as a model.

This study used the weight drop model for TBI [14,18]. We assessed the impact of the 2-Cl-MGV-1 treatment on TBI and on motor behavioral deficits resulting from the TBI. To this end we examined changes in MRI and in tissue over the 60-day period following the TBI, as well as using a treadmill apparatus to monitor effects on neurological motor-function.

Our study found that within 22 days after induction of TBI, 2-Cl-MGV-1-treated mice learned to avoid foot shocks on the treadmill apparatus and reached performance levels comparable to naïve mice.

The MRI showed that 60 days of 2-Cl-MGV-1 administration reduced the size of the TBI-damaged tissue significantly. This beneficial effect may have been achieved by the ability of 2-Cl-MGV-1 to reduce edema and bleeding in the TBI area [5]. It also appears that following the 60 days of 2-Cl-MGV-1 administration, the tissue regenerated almost to its normal state, and the Nissl staining showed a normal level of neurons in the injured area. Microglia activation (gliosis) was not conspicuous after the 60-day period, suggesting that active inflammatory response is reduced.

**Figure 8:**
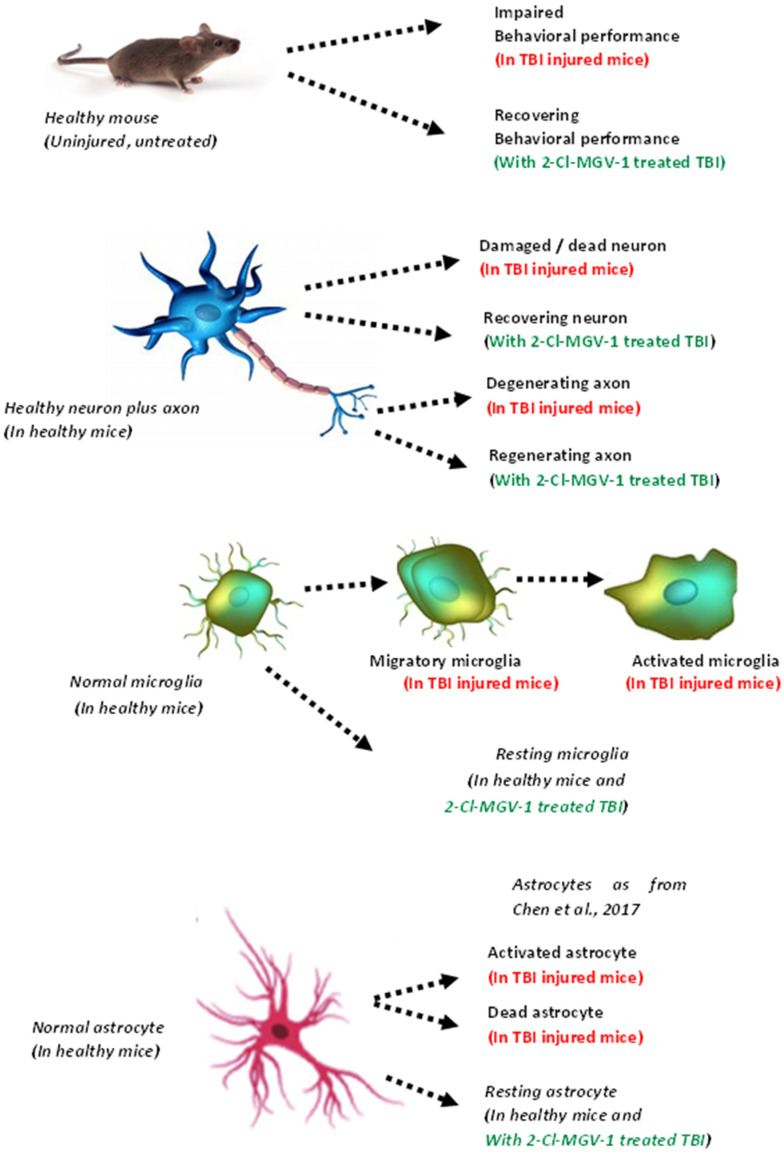
Top row: Mouse, behavioral performance, healthy mouse compared to TBI injured mouse with and without 2-Cl-MGV-1 treatment. The 2-Cl-MGV-1 restores normal behavior. Second row: Undamaged neuron and axon in healthy mouse compared to neuron and axon damaged by TBI with and without 2-Cl-MGV-1 treatment. The 2-Cl-MGV-1 restores the neurons to healthy morphology. Third row: Normal microglia (resting) in healthy mouse compared to microglia affected by TBI (i.e., becoming migratory and activated microglia). The 2-Cl-MGV-1 reduces the level of activation of microglia. Fourth row: Healthy astrocyte in healthy mice versus astrocytes affected by brain injury. Treatment with 2-Cl-MGV-1 counteracts the effects of brain injury on astrocytes [14]. Summarizing, TBI impairs neuro-behavior in mice, has injurious effects on neurons, and activates microglia. These adverse effects are counteracted by 2-Cl-MGV-1 treatment. The effects on astrocytes need further study.

The complex mechanisms involved in the therapeutic effects of 2-Cl-MGV-1 in TBI are illustrated in Figure 8 below.

### 3.1. Comparison of 2-Cl-MGV-1 Efficacy with Other TSPO Ligands

It is well known that neuroinflammation and cell death are among the common sequela of various brain diseases and injuries [5,19,20,21]. Previous studies suggest that brain damage can be attenuated by TSPO ligands such as Ro5 4864, PK 11195, 2-Cl-MGV-1, Etifoxine, and Emapunil [5,16,17,21]. Other studies show that naturally occurring compounds that bind to TSPO, such as retinoic acid and curcumin, may also have beneficial effects on brain damage [22,23,24]. Our previous studies suggest that the simultaneous actions of TSPO ligands on mitochondria (modulating ATP, ROS, ΔΨm, and Ca^2+^ equilibria), cell nucleus (modulating gene expression, including immediate early genes), synthesis of neurosteroids and neuroinflammation may be involved in the pathways whereby TSPO ligands can counteract neurodegeneration in the brain [5,6,10,16,17,25,26,27].

### 3.2. Other Neurological Disorders Where 2-Cl-MGV-1 May Be Beneficial

In a previous study in a transgenic mouse model of Huntington’s disease, we demonstrated that 2-Cl-MGV-1 can prolong the life span of mice [10]. It is thus possible that our current findings regarding TBI have broader implications for protection of the brain from harmful neurodegenerative processes.

## 4. Materials and Methods

### 4.1. Mouse Description

This study included 152 male *C57BL/6N* mice (Harlan, Jerusalem) aged 6–15 weeks (6–7 weeks at the start of the study), whose weight range was 20–24 g at the time of TBI induction. We chose mice as a model because 2-Cl-MGV-1 was previously shown to have protective effects in a transgenic mouse model of Huntington’s Disease [10,28]. Mice were kept in individually ventilated cages (up to 6 mice per cage) at 20–23 °C under a 12 h light/dark cycle with free access to water and food. Before starting experimental procedures, the mice were acclimatized for a week to the specific pathogen-free animal facilities.

The mice’s age range was chosen to ensure that the mice were in the young adult stage, which is ideal for investigating neurobiological processes, as this period is characterized by stable physiological development and high neural plasticity. This age range has been widely used in neurobiology research because it minimizes the potential influence of age-related degeneration and health complications, providing reliable data on the biological processes being studied [10,28].

The study was approved by the Technion committee for experiments in animals (IL-034-03-2015).

### 4.2. Study Design

The final sample size for each experimental group was determined using a set-based approach to ensure adequate statistical power and balanced injury severity across groups. By using repeated experimental sets and ensuring equal distribution of injury severity based on MRI findings, as described below, we were able to obtain a final sample size of at least 5 mice per group with balanced injury severity, ensuring that the results were both statistically valid and reflective of the intended experimental conditions.

The mouse model (C57BL/6N) used for TBI induction is a standard one for such purpose, and TBI was induced using the weight drop model.

In Experiment 1, the mice were divided randomly into a 2-Cl-MGV-1 (+DMSO as a vehicle) treatment group and a vehicle only (DMSO) treatment group. In Experiment 2, the mice were assigned to either the 2-Cl-MGV-1 or the DMSO group so that the initial TBI size, as assessed by MRI, was divided equally between the two groups. In Experiment 3, assignment to TBI groups was also determined by TBI size, but here, there were 3 TBI-groups.

Mice were treated after TBI with either the low-affinity TSPO ligand 2-Cl-MGV-1 or with DMSO vehicle.

The first treatment after TBI was administered within a time window of 3–11 h. This was completed in order to evaluate potential clinical applicability (i.e., time to reach care facility, etc.).

Ligand and vehicle treatments were administered daily (except in Experiment 1, when Fridays and Saturdays were skipped) for a total period of 59 days.

The changes, over time, in brain injury volume were monitored with MRI.

Behavioral studies were performed in order to evaluate deficits in motor capabilities following TBI.

Brain damage status was evaluated after the 59-day treatment period by examining neuroanatomical, neurohistological, and immunohistochemical status.

The safety of 2-Cl-MGV-1 was assessed by examining the liver and kidney function of mice that did not undergo TBI, after a 59-day treatment period with daily 2-Cl-MGV-1 or vehicle injections.

All assessments were conducted in a double-blind manner.

### 4.3. Tool Description

#### Weight Drop Apparatus for Induction of TBI

Blunt traumatic brain injury was induced in the mice with a Shohami’s weight drop apparatus [29], which a previous study showed to mimic human TBI conditions, including the variability from individual to individual [18,30].

Focal injury was induced in the left hemisphere of the mice with a Teflon tipped, round, 2 mm diameter cone that was placed on the exposed skull, 1 mm lateral to the midline and 1 mm caudal to bregma. A 95 g weight was then dropped onto the cone through a Plexiglas tube, from a height of 6 cm.

Immediately prior to the procedure, the animals were anesthetized with 0.5–1.5% isoflurane (Piramal Critical Care, Bethlehem, PA, USA) supplemented with oxygen (0.8 L/min), applied by a Midmark VME2 Wall Mount System anesthesia machine UXR, (Pointe-Claire, QC, Canada). In Experiments 2 and 3, we used a sponge cushion to stabilize the position of the mice’s heads during the TBI procedure (lesson learned from Experiment 1). The mice were carefully monitored, and if necessary, mechanically stimulated to ensure continuous breathing.

### 4.4. Magnetic Resonance Imaging (MRI)

An Aspect M2™ Compact High-Performance MRI scanner for rodents (Aspect Imaging, Shoham, Israel) was used for imaging the mouse brains. This scanner is a compact 1 Tesla micro-MRI system used for in vivo imaging of small animals (rodents). The MRI apparatus is equipped with a cylindrical radiofrequency head coil for signal excitation and reception. During imaging, the mouse was under anesthesia of 0.5–1.5% isoflurane, supplemented with oxygen (0.8 L/min), as described above. The animal’s respiration was continuously monitored with the Midmark VME2 Wall Mount System, which was used to record a coronal T2-weighted scan across the whole brain for each animal: Fast Spin Echo (FSE) sequence slice thickness = 0.8 mm, slice gap = 0.1 mm, FOV = 4 × 4 cm, matrix dimension = 200 × 200, spatial resolution = 200 × 200 μm^2^, repetition/echo time (TR/TE) = 3000/80 ms, number of excitations = 4, number of averages = 4.

#### 4.4.1. Calculation of the MRI Signal

MRI analysis was performed using the same intervals throughout the study. The signal intensities of all the regions of interest (ROIs) in the 0.8 mm-thick brain sections were evaluated for each brain scan, both in the TBI-impacted hemisphere (left hemisphere) and the contralateral hemisphere (right hemisphere). T2-weighted MRI image analyses were applied at the site of the injuries. The MRI images showed white areas (hot spots) related to edema or bleeding and black areas (lesions) related to tissue damage (hole). The size of the injury was quantified with MATLAB R2014a (v. 8.3.0.532, MathWorks, Natick, MA, USA) and compared between the ligand treatment group and the vehicle treatment group. Focal lesions were identified as hot spots (white areas) or lesions (black areas) by visual inspections of the MRI.

#### 4.4.2. Treadmill

The mice’s motor capacity was assessed using a treadmill apparatus (Rodent Treadmill NG, Cat. No. 47300, UGO Basile S.R.L, Gemonio, Italy). The treadmill is an exercise machine using rolling belts and a shock grid. The speed and inclination of the belts is adjustable, and the observer chooses the duration of the test. During the test, the shock grid delivers a mild electric shock whenever the mouse stops in order to encourage it to continue moving.

The parameters that were measured included

- The number of shocks an animal received (where the higher the number of shocks, the more severe the motor impairment)
- The maximal running distance (where the longer the distance, the less the motor impairment).

### 4.5. Treatment

Mice were treated with injections of vehicle (DMSO) alone or 2-Cl-MGV-1 in vehicle.

A dose of 7.5 mg/kg of 2-Cl-MGV-1 in vehicle (DMSO) was injected subcutaneously in the back neck area of the mice within a window of 3 to 11 h after TBI induction, and this treatment was repeated daily, 5 days/week in Experiments 1 and 4 or 7 days/week in Experiments 2–4, for a total period of 59 days. Vehicle alone was injected the same number of times as the ligand.

### 4.6. Sacrifice of Mice and Fixation of Brains

At the end of the 59-day treatment period, animals were fully anesthetized with ketamine/xylazine (10:1 mg/kg i.p.) and perfused via the left heart ventricle with phosphate-buffered saline (PBS, pH = 7.4, 10 mL) followed by 4% paraformaldehyde in PBS (4% PBS for 10 min). Speed of perfusion was 15 mL/min.

Next, the mouse brains were extracted and dehydrated in a series of ethanol (70%, 80%, 96%), and 2× isopropanol (100%). Following clearing in chloroform, the brains were embedded in paraffin and stored until ready to be cut or sent away.

### 4.7. Experiments on Traumatic Brain Injury and Safety of 2-Cl-MGV-1 Ligand

The 2-Cl-MGV-1 dosage used in all experiments was 7.5 mg/kg and was based on previously published studies [10,14].

Brain imaging (MRI) and behavioral tests (treadmill) were used to evaluate the brain damage resulting from the TBI, and blood serum tests were used to assess 2-Cl-MGV-1 safety.

#### 4.7.1. Experiment 1 (Preliminary)

##### Primary Goal: To Test Feasibility

**Figure 9:**
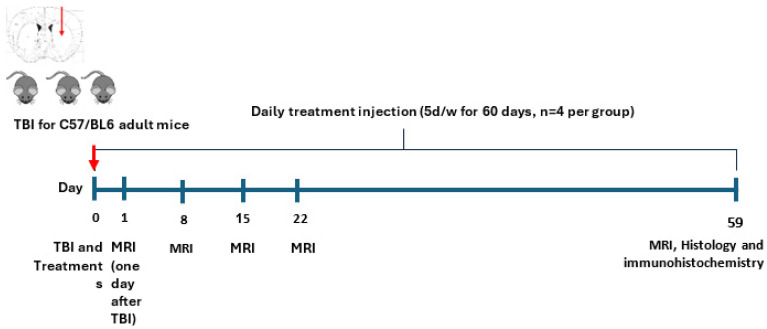
(Experiment 1): Timeline of the preliminary experiment showing MRI. Included 2 groups: (1) TBI + vehicle—DMSO-treated TBI mice; (2) TBI + ligand—2-Cl-MGV-1-treated TBI mice. TBI—traumatic brain injury; treatment = vehicle or 2-Cl-MGV-1, 3–7 h after TBI, then daily Sunday–Thursday, for 59 days. MRI days 1, 8, 15, 22, and again on day 59.

This preliminary experiment aimed to assess the effect of 2-Cl-MGV-1 on TBI. Eight male *C57BL/6N* mice (body weight 20–24 g) were divided into 2 groups (4 mice in each): (1) TBI + Vehicle: DMSO (vehicle) treated TBI mice; (2) TBI + Ligand: 2-Cl-MGV-1 (ligand + vehicle) treated TBI mice. MRI was performed one day after the TBI induction (day 1) and then repeated on days 8, 15, and 22, and a final MRI was conducted on day 59. In this experiment, treatments started 3–7 h after induction of TBI. The treatment regime thereafter consisted of one injection per day, 5 days per week (starting on day 1 after TBI and not on Fridays and Saturdays) over a period of 59 days (Figure 9).

##### Histological Evaluation of Brain Damage in Experiment 1

After 59 days of treatment, the mice were sacrificed and their brains fixated as previously described. The brains were then cut into 5 µm coronal sections on a microtome. The sections that included the injured areas were deparaffinized for 5 min in xylene, followed by 2 × 5 min in 100% ethanol and were captured on glass slides.

For Nissl staining of the sections, 0.1% cresyl violet in acetate buffer was applied for 30 min, then the sections were rinsed in tap water and dehydrated in 70% ethanol. Intensity of staining was evaluated microscopically. The sections were further dehydrated 2 × 3 min in 100% ethanol, cleared in xylene, and cover-slipped with dibutylphthalate polystyrene xylene (DPX) mounting medium (Fluka, BioChemika, Munich, Germany).

After two days of hardening in DPX, the Nissl-stained sections were studied microscopically for histological identification of damaged brain areas and morphological changes following injury as well as for the treatment effects. The brain regions of interest were captured with a VM microscope, panoramic 250 Flash III (3DHistech, Budapest, Hungary) at the Image Analysis Section of the Biomedical Core Facility of the Rappaport Family Institute for Research in the Medical Sciences, Ruth and Bruce Rappaport Faculty of Medicine, The Technion, Haifa, Israel.

#### 4.7.2. Experiment 2

Primary goals: Further assessment of the effects of 2-Cl-MGV-1 (7.5 mg/kg) on mice that underwent TBI.

This experiment included 12 *C57BL/6N* male mice whose body weight was 20–24 g and was based on the learnings from Experiment 1 above. We cushioned the mouse head with a sponge, which stabilized the position of the head in its various dimensions (up down, left right, forward backward). In the weight drop apparatus, we reduced the play between the stopper and the drop tube to increase the precision of the impact site.

**Figure 10:**
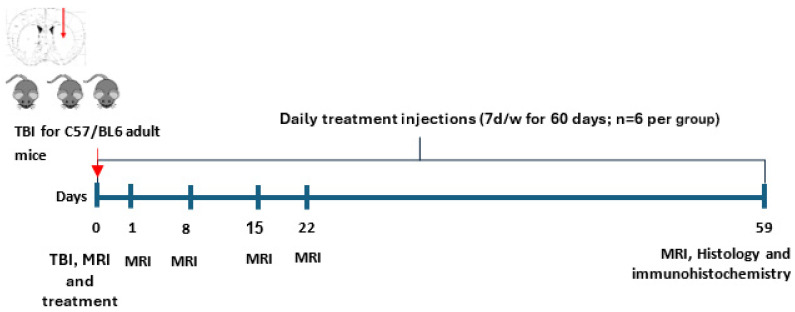
(Experiment 2): Timeline for MRI monitoring. TBI—traumatic brain injury, both groups (2-Cl-MGV-1 and vehicle); treatment = 2-Cl-MGV-1 or vehicle, 7–11 h after TBI, then daily for 59 days. MRI—on days 0 (day of the TBI), then weekly on days 1, 8, 15, 22, and again on day 59.

MRI was conducted 2–3 h after TBI induction (day 0). Next, the mice were divided into a ligand (2-Cl-MGV-1) treatment group (TBI + ligand) and a vehicle (DMSO) treatment group (TBI + vehicle) as in Experiment 1. In this experiment, however, we ensured that the various injury sizes (as observed in the MRI) were equally distributed between the two groups. Each group contained 6 mice. Treatment with 2-Cl-MGV-1 or vehicle started 7–11 h after TBI and was thereafter administered daily, 7 days per week, for a period of 59 days. MRI was repeated on days 1, 8, 15, 22, and 59 after the TBI day. All the mice were sacrificed after day 59, and their brains were dissected and prepared for neuroanatomical cytochemistry and histological analyses (Figure 10).

#### Histological Evaluation of Brain Damage in Experiment 2

After sacrifice and fixation were conducted as described earlier, the fixated brains were sent to a laboratory at the University of Oviedo, Spain. There, the brains were dissected into blocks along four different planes, resulting in five blocks per brain. Each block was placed in a separate biopsy cassette for processing and paraffin embedding. The blocks were then dehydrated with a series of ethanol at graded concentration (50%, 70%, 80%, 96%, and 100%) and cleared twice in isopropanol (100%) before being embedded in paraffin. Ten µm coronal sections were cut with a rotary microtome (LeicaRM2155, Leica Biosystems Nussloch GmbH, Nussloch, Germany) from each paraffin-embedded brain block. Brain sections, including those from the injured areas, were captured on Superfrost^®^ Plus microscope glass slides (Thermo Scientific, Waltham, MA, USA). For basic staining with hematoxylin/eosin, the brain sections were deparaffinized thoroughly using xylene, rehydrated with a series of ethanol at graded concentrations (100%, 96%, 70% and 50%)—2 min at each concentration, and then rinsed twice in distilled water. Sections were then stained with 0.1% Mayer’s hematoxylin for 7 min, rinsed for 5 min in tap water, and counterstained with 0.25% eosin for 1 min, followed by rinsing in distilled water. After staining, brain sections were dehydrated in a series of ethanol at graded concentrations (70%, 96%, and 100%) for 1 min at each concentration and rinsed in xylene for at least 2 min. The glass slides were cover-slipped with Eukitt^®^ Quick-hardening mounting medium for microscopy (Sigma-Aldrich, Burlington, MA, USA).

Nissl staining of the sections was conducted according to the protocol described by Navarro et al. [31]. The deparaffinized and rehydrated brain sections were incubated for 2 min in a solution of 0.01% thionin, 0.01% cresyl violet, and 0.05% methyl green, rinsed twice for 1 min in distilled water and twice for 1 min in 96% ethanol. The samples were dehydrated twice for 1 min in 100% ethanol, rinsed in xylene for at least 3 min, and then cover-slipped with Eukitt^®^ Quick-hardening mounting medium for microscopy. The Eukitt cover-slipped sections were allowed to harden for at least 24 h prior to microscopic evaluation.

#### Preparation of Brain Sections for Immunohistochemical Analyses in Experiment 2

For enhancement of immunostaining, representative deparaffinized and rehydrated brain sections, captured on Superfrost^®^ Plus microscope glass slides, were subjected to antigenic retrieval by automatized incubation for 20 min at 95 °C, in a buffer of pH 6 or 9 (depending on the primary antibody). After 5 min at room temperature, the brain sections were permeabilized for 10 min in 0.01% triton/PBS and rinsed 3 times in PBS. In order to block endogenous peroxidase activity, brain sections were treated with 3% (w/v) H_2_O_2_ in methanol for 20 min and then rinsed 3 times in PBS. Sections were then incubated for 30 min in normal serum, diluted 1:30 in PBS from the same species in which the secondary antibody was raised. Incubations with specific primary antibodies, appropriately diluted in PBS, were conducted overnight at 4 °C in a humid and dark chamber, as follows: 1/100 β-III-Tubulin (T2200, Sigma-Aldrich, Burlington, MA, USA), 1/50 NeuN/RBFOX3 (SAB4301175, Sigma-Aldrich, Burlington, MA, USA), and 1/200 Iba-1/AIF1 (MABN92, Sigma-Aldrich, Burlington, MA, USA). After rinsing 3 times in PBS, brain sections were incubated with specific horseradish peroxidase (HRP)-conjugated secondary antibodies, at the recommended dilution (1/500–1/1000, depending on each specific antibody) for 90 min at room temperature in a humid and dark chamber and were then rinsed 3 times in PBS. Increased signal intensity was achieved through the incubation of brain sections with 1/200 Peroxidase-Anti-Peroxidase-Soluble Complex (PAP; Sigma-Aldrich, Burlington, MA, USA) for 1 h at room temperature in a humid and dark chamber. Next, 3,3′-Diaminobenzidine (DAB; Sigma-Aldrich, Burlington, MA, USA) substrate for HRP-conjugated secondary antibodies was added for 10 min followed by rinsing at least once in tap water. Counterstaining with hematoxylin was applied for 7 min, as described above. After these immunohistochemical preparations, the sections were cover-slipped with Eukitt^®^ quick-hardening mounting medium for microscopy. Sections prepared as hereby described were allowed to harden for at least 24 h.

#### Microscopy and Image Quantification in Experiment 2

Next, brain sections were studied microscopically for histological identification of the damaged brain areas, for anatomical changes following injury, and for the effects of treatment with 2-Cl-MGV-1. The brain areas of interest, namely those that were close to the brain damage, were captured with a Nikon EClipse E400 microscope equipped with a Nikon DS-Fi1 camera at the Department of Morphology and Cell Biology, Faculty of Medicine, University of Oviedo, Oviedo, Spain. Quantification of cells positive for each specific stain was conducted in 3 different fields that were close to the area of the brain damage. Data are presented as mean ± SEM, with significance set at *p* < 0.05. Unpaired *t*-test was used to evaluate the effect of 2-CI-MGV-1 treatment on TBI brains and compared to TBI brains treated with vehicle (DMSO).

#### 4.7.3. Experiment 3

##### Primary Goals: Assessment of the Protective Effect of 2-Cl-MGV-1 Treatment on TBI Behavioral Outcomes

This experiment used a new mouse cohort. The mice weighed 17–20 g on day 0 of the experiment (i.e., TBI day). The learnings from Experiment 1 were used in this experiment, as was the case in Experiment 2. Thus, the mouse heads were cushioned, and following MRI on the TBI day, the mice were divided into groups so that all groups included a similar distribution of injury sizes. The mice that had undergone TBI were divided into three groups as follows: (1) TBI untreated (TBI without any treatment; *n* = 10); (2) TBI + vehicle (TBI with DMSO treatment; *n* = 7); (3) TBI + ligand (TBI treated with 2-Cl-MGV-1; *n* = 10). In addition, there was a fourth group—the naïve group (*n* = 5): mice that neither underwent TBI nor received any treatment but did participate in the assessments. MRI of the injury size was repeated on days 1, 8, 15, 22, and 59 following the TBI day. The first injection of 2-Cl-MGV-1 or vehicle was administered to the appropriate groups, between 4.5 and 5.5 h after induction of TBI. Motor and learning performance, which are indicative of impairments and recuperation of the mice, were assessed with the aid of the treadmill apparatus on days 1, 8, 15, and 22 following TBI induction. On these days, injections of vehicle and treatment were always administered after the behavioral assessment in order to avoid the effect of the stress associated with injection.

##### Statistical Analysis

Statistical analysis was performed using GraphPadPrizm^®^ software, version 10 (La Jolla, CA, USA). Repeated measures analysis of variance (RM-ANOVA) was applied followed by Bonferroni, Dunnet, or Mann–Whitney post hoc test as appropriate. Unpaired *t*-test was applied as appropriate. Results are expressed as mean ± SEM, and *p* < 0.05 was considered statistically significant. In all groups in all experiments *n* ≥ 4.

#### 4.7.4. Experiment 4

Primary goals: To assess the safety of 2-Cl-MGV-1 administration as reflected by liver and kidney functions.

This experiment included 30 C57BL/J male mice that had not undergone TBI, to assess the safety of 2-Cl-MGV-1 as compared to the vehicle DMSO and to naïve mice. This experiment included 5 groups (*n* = 6 in each group): (1) naïve without any treatment; (2) daily vehicle (DMSO) injections administered 5 days per week for a total period of 59 days; (3) daily 2-Cl-MGV-1 injections administered 5 days per week for a total period of 59 days; (4) daily vehicle (DMSO) injections administered 7 days per week for 59 days; (5) daily 2-Cl-MGV-1 (in vehicle) injections administered 7 days per week for 59 days. After 59 days, the mice were anesthetized with ketamine and xylene and then blood was taken from the heart by a syringe without needle (to avoid hemolytic reaction). Then, the blood was centrifuged to separate the serum from the cells, and liver and kidney tests were performed according to the manufacturer’s instructions (Architect, Core Laboratory, Abbott, Lake Forest, IL, USA). The tests included aspartate aminotransferase (AST), alanine aminotransferase (ALT), alkaline phosphatase, direct bilirubin, total bilirubin, urea nitrogen, and creatinine and were conducted at Dr. Marlen Kaplan’s Biochemical Laboratory at the Rambam Medical Center in Haifa.

## 5. Conclusions

It seems that TBI in a mouse model can be attenuated by treatments that target mitochondrial activity and nuclear gene expression, as was demonstrated by the TSPO ligand 2-Cl-MGV-1 and was reflected in brain imaging and histological and behavioral assessments. The relevance of these findings to neurodegenerative processes is as yet unclear. Comparative studies among various candidates for the treatment of TBI and other brain diseases merit further investigation.

## Data Availability

The original contributions presented in this study are included in the article. Further inquiries can be directed to the corresponding author.
